# Autophagy-Related Genes and Long Noncoding RNAs Signatures as Predictive Biomarkers for Osteosarcoma Survival

**DOI:** 10.3389/fcell.2021.705291

**Published:** 2021-08-26

**Authors:** Jian Zhang, Rui Ding, Tianlong Wu, Jingyu Jia, Xigao Cheng

**Affiliations:** ^1^Department of Orthopedics, The Second Affiliated Hospital of Nanchang University, Nanchang, China; ^2^Institute of Orthopedics of Jiangxi Province, Nanchang, China; ^3^Institute of Minimally Invasive Orthopedics, Nanchang University, Nanchang, China

**Keywords:** osteosarcoma, autophagy, lncRNA, prognostic signature, biomarkers

## Abstract

Osteosarcoma is a common malignant tumor that seriously threatens the lives of teenagers and children. Autophagy is an intracellular metabolic process mediated by autophagy-related genes (ARGs), which is known to be associated with the progression and drug resistance of osteosarcoma. In this study, RNA sequence data from TARGET and genotype-tissue expression (GTEx) databases were analyzed. A six autophagy-related long noncoding RNAs (ARLs) signature that accurately predicted the clinical outcomes of osteosarcoma patients was identified, and the relations between immune response and the ARLs prognostic signature were examined. In addition, we obtained 30 ARGs differentially expressed among osteosarcoma tissue and healthy tissue, and performed functional enrichment analysis on them. To screen for prognostic-related ARGs, univariate and LASSO Cox regression analyses were successively applied. Then, multivariate regression analysis was used to complete construction of the prognostic signature of ARGs. Based on the risk coefficient, we calculated the risk score and grouped the patients. Survival analysis showed that high-risk patients evolve with poor prognosis. And we verified the prognosis model in the GSE21257 cohort. Finally, verification was conducted by qRT-PCR and western blot to measure the expression of genes. The results show that autophagy-related marker models may provide a new therapeutic and diagnostic target for osteosarcoma.

## Introduction

Osteosarcoma is a common and extremely threatening bone malignant tumor, occurs usually in adolescents, and children (Kansara et al., 2014). The current treatment methods include surgical resection and chemotherapy before and after surgery, for the patients with non-metastatic osteosarcoma, the 5-year survival rate of can reach 70% (Isakoff et al., 2015). However, about the patients with metastatic or recurrent osteosarcoma, the 5-year overall survival rate is only 20%, and has changed little over the past 30 years (Goorin et al., 2003; Kempf-Bielack et al., 2005; Meyers et al., 2011). In addition, osteosarcoma often develops resistance to standard chemotherapy regimens, which seriously impacts patient prognosis (Brown et al., 2017; Lilienthal and Herold, 2020). Therefore, seeking potential targets for effective osteosarcoma therapy is particularly important and necessary.

Autophagy is the main intracellular metabolic degradation process modulated by autophagy-related genes (ARGs), autophagosomes phagocytose cytoplasmic materials, then degradation, and recycling by lysosomes (Mizushima and Komatsu, 2011; Boya et al., 2013). In cancer, autophagy can exert two functions, either inhibitory and stimulatory actions, the specific role depends on the occurrence, progression, and type of cancer (Nazio et al., 2019; Mizushima and Levine, 2020). Deletion of the autophagy gene BECN1 is common in breast, ovarian, and prostate cancers (Kondo and Kondo, 2006). In various mouse models, the systemic or tissue-specific deletion of genes essential for autophagy can lead to defects in autophagy, and which accelerates the occurrence of tumors. For example, *becn1*^+/–^ mice spontaneously develop malignant tumors, including lymphoma, lung cancer, and liver cancer (Liang et al., 1999; Qu et al., 2003). Autophagy also affects certain cellular processes, such as the epithelial to mesenchymal transition or migration, and therefore inhibits tumor progression and metastasis (White et al., 2015; Li et al., 2020). In short, autophagy can promote or inhibit cancer progression and metastasis at different stages of the disease. The link between cancer and autophagy is complex and requires further research.

Long noncoding RNAs (lncRNAs) have many functions (Ulitsky and Bartel, 2013). LncRNA is not only involved in the normal biological functions, but also participate in the process of a variety of diseases, including those related to tumor occurrence, development, and metastasis (Martens-Uzunova et al., 2014; Bartonicek et al., 2016). There is ample evidence that lncRNAs mediate the transcription and post-transcriptional levels of ARGs to regulate tumor development (Chen J.F. et al., 2018; Cai et al., 2019; Xu et al., 2019). However, there has been no systematic evaluation of the characteristics of ARGs in patients with osteosarcoma and their correlation with overall survival. Therefore, the purpose of our study was to identify new autophagy-related markers for the diagnosis and prognosis of osteosarcoma.

In this research, we identified a six autophagy-related lncRNAs (ARLs) signature has accurate predictive properties for the outcome of osteosarcoma patients. Then, we explored the role of the immune responses in the prognosis of autophagy. We also identified three ARGs that showed good performance for predicting the clinical outcomes of osteosarcoma patients. In a separate dataset (GSE21257), we evaluated their accurate prediction for the prognosis of patients with osteosarcoma. Our results show that autophagy-related marker models may provide potential prognostic and diagnostic targets for osteosarcoma therapy.

## Materials and Methods

### Data Collection and Autophagy-Related Genes

The RNA expression data of healthy tissues were collected from the genotype-tissue expression database.^1^ The fragments per kilobase of transcript per million mapped reads (FPKM) values in the expression data were converted using log2(*x* + 0.001). The RNA sequence data and osteosarcoma patient characteristics were collected from the TARGET database.^2^ TARGET and GTEx data sets were merged and batch-to-batch variation was removed in the “sva” R package. The FPKM values were used to indicate gene expression levels.

A microarray data set, named the GSE21257 data set, was obtained by downloading the gene expression data of 53 patients with osteosarcoma and their corresponding clinical information from the gene expression omnibus database.^3^ Gene expression levels are shown as normalized signal values. The Human Autophagy Database^4^ was used to obtain ARGs.

### Differentially Expressed ARGs and Functional Annotation

LogFC > 1 and *p* < 0.05 as the threshold, differentially expressed ARGs (DE-ARGs) and lncRNAs in osteosarcoma, and healthy tissues were identified using the limma package. gene ontology (GO) and kyoto encyclopedia of genes and genomes (KEGG) pathway analyses were performed for the DE-ARGs. The construction and visualization of the protein-protein interaction (PPI) network diagram are implemented by online tools STRING and Cytoscape software.

### Identification and Construction of Prognostic Signatures

By univariate Cox regression analysis, we determined the ARGs that were associated to the survival in osteosarcoma patients from TARGET dataset (*p* < 0.01). Next, we used LASSO Cox regression analysis to filter the best prognostic genes. Finally, a prognostic signature was established by multivariate Cox regression analysis, which composed of three ARGs.

In order to obtain ARLs, in the TARGET database, we calculated the Pearson correlation coefficient to determine the correlation between the expression levels of lncRNAs, and ARGs. With the absolute value of the correlation coefficient greater than 0.4 (| R| > 0.4) and the *P*-value less than 0.05 (*P* < 0.05) as the standard, ARLs was screened out. Subsequently, to construct an ARLs prognosis model, we selected the differentially expressed ARLs, applied univariate Cox regression to screen for prognostic ARLs, and then constructed a ARLs prognostic signature including six ARLs by LASSO Cox analysis. The following formula was used to calculated risk score of each patient:

$$R\,i\,s\,k\,s\,c\,o\,r\,e=\sum_{i=1}^{n}\left(C\,o\,e\,f_{i}*x_{i}\right)$$

the *Coef_i_* means risk coefficients, *x_i_* is the ARGs or ARLs expression value.

### Evaluation and Verification of the Prognostic Signature

We rank the risk scores of all patients (calculated by the above formula), take the median value, and divide the patients based on this. Using Kaplan–Meier curve, we distinguished the survival of the two groups of patients. Based on the receiver operating characteristic (ROC) curve, we evaluated the prediction effectiveness of the signature. We also performed univariate and multivariate Cox regression analysis to assess whether the risk score was independent of other clinical variables, including age, sex, and metastasis. Subsequently, according to the clinical characteristics of patients, subgroup analyses were performed for individual genes in the autophagy-related prognostic models. Then, according to the patient’s clinical information and risk, we used the “rms” package to establish a nomogram for clinical evaluation. A calibration curve was drawn to evaluate the agreement between the actual clinical outcome and the predicted clinical outcome, and the C index was calculated for the nomogram model. An independent dataset (GSE21257) was used to testify the reliability of the nomogram model and ARGs prognostic signature.

### Gene Set Enrichment Analysis and Single Sample GSEA

Gene set enrichment analysis (GSEA) software (version 4.1.0) was used to evaluate the functional phenotype between the high-risk group and the low-risk group based on the ARLs prognostic signature. GO gene sets (go.bp.v7.4.symbols.gmt) were downloaded from Molecular Signatures Database as the reference gene set (Subramanian et al., 2005). Nominal *p*-value < 0.05 and NES (Normalized Enrichment Score) > 2 were set as the cut-off. ssGSEA was used to evaluate differential expression of immune cell infiltration and immune-related functions between the two groups of patients.

### Cell Lines and Cell Cultures

The human osteoblast cell line hFOB1.19 and two human osteosarcoma cell lines (U2OS and 143B) were purchased from the National Collection of Authenticated Cell Cultures (Shanghai, China). The cells were cultured in Dulbecco’s modified Eagle’s medium (DMEM, Gibco) containing 10% fetal bovine serum (FBS, Gibco) and 1% penicillin/streptomycin (Thermo Fisher Scientific, United States). The human osteoblast cell line hFOB 1.19 was cultured at 34°C with 5% CO_2_, and the osteosarcoma cell lines were cultured at 37°C with 5% CO_2_ in a humid atmosphere.

### RNA Extraction and Reverse-Transcription Quantitative PCR

Total RNA of cells was extracted with TRIzol reagent (Invitrogen, United States), and reverse transcribed into cDNA with PrimeScript^TM^ RT reagent Kit (Takara, Japan). Reverse-transcription quantitative PCR (RT-qPCR) was performed with SYBR Green qPCR Master Mixes (Thermo Fisher Scientific, United States). Glyceraldehyde 3-phosphate dehydrogenase (GAPDH) was used as the normalization control for lncRNA and mRNA. Primer information is provided in Supplementary Table 1.

### Western Blot

To extract total protein from cells, RIPA lysis buffer (Beyotime, China) was used. To quantify proteins, a BCA protein quantification kit (Beyotime, China) was used. Proteins were separated on SDS-polyacrylamide gel and transferred to a PVDF membrane (Millipore, United States). The membrane was blocked with skim milk, incubated with the primary antibody overnight at 4°C, and incubated with the secondary antibody for 2 h at room temperature. The antibodies used in this study were AMBRA1 (1:500, Proteintech, China), MYC (1:1000, Abcam, United Kingdom), VEGFA (1:1000, Abcam, United Kingdom), GAPDH (1:5000, Abcam, United Kingdom), and goat anti-rabbit secondary antibody (1:5000; Abcam, United Kingdom). The protein bands were quantified using Image J software (version 1.8.0), and GADPH was used as the loading control.

### Statistical Analysis

R v4.0.4 and GraphPad Prism v9.2 were used for the statistical analysis. Student’s *t*-test was used to compare differences between two groups. Differences were considered to be significant at *p* < 0.05 if not specified.

## Results

### Identification of Autophagy-Related lncRNAs and Signature Establishment

**FIGURE 1:**
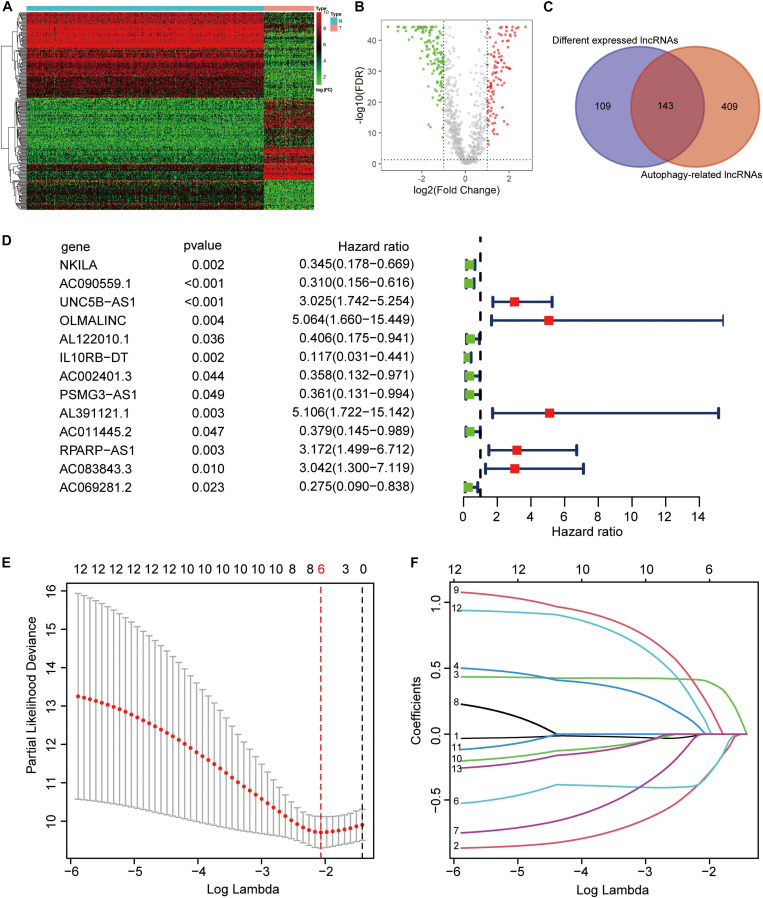
Construction of autophagy-related lncRNA signature. **(A)** Heatmap of the differentially expression long noncoding RNAs (lncRNAs). **(B)** Volcano plot. **(C)** Venn diagram. **(D)** Univariate cox regression showed 13 lncRNAs related to osteosarcoma survival. **(E,F)** Lasso regression for lncRNAs in univariate cox regression. Coefficients refers to the risk coefficient corresponding to each gene.

From the TARGET (*n* = 84) and GTEx (*n* = 396) RNA sequence data, 222 ARGs, and 994 lncRNAs were extracted. Based on the parameters *p* < 0.05 and logFC ≥ 1, we identified 252 different expressed lncRNAs (DELs). The heatmap and volcano plot showed DELs (Figures 1A,B). Then, by calculating the Pearson correlation coefficient between the expression of the ARGs and lncRNAs, using | R| > 0.4 and *p* < 0.05 as the selection criteria, and we obtained 552 ARLs. As shown in the Venn diagram demonstrated that there were 143 lncRNAs in the intersection of DELs and ARLs (Figure 1C). After univariate Cox regression analysis of the 143 lncRNAs, 13 prognostic ARLs were obtained (Figure 1D). Last, further LASSO Cox regression analysis determined the prognostic signature, which comprised six ARLs (Figures 1E,F and Supplementary Table 2).

### Evaluation of the ARLs Prognostic Signature

**FIGURE 2:**
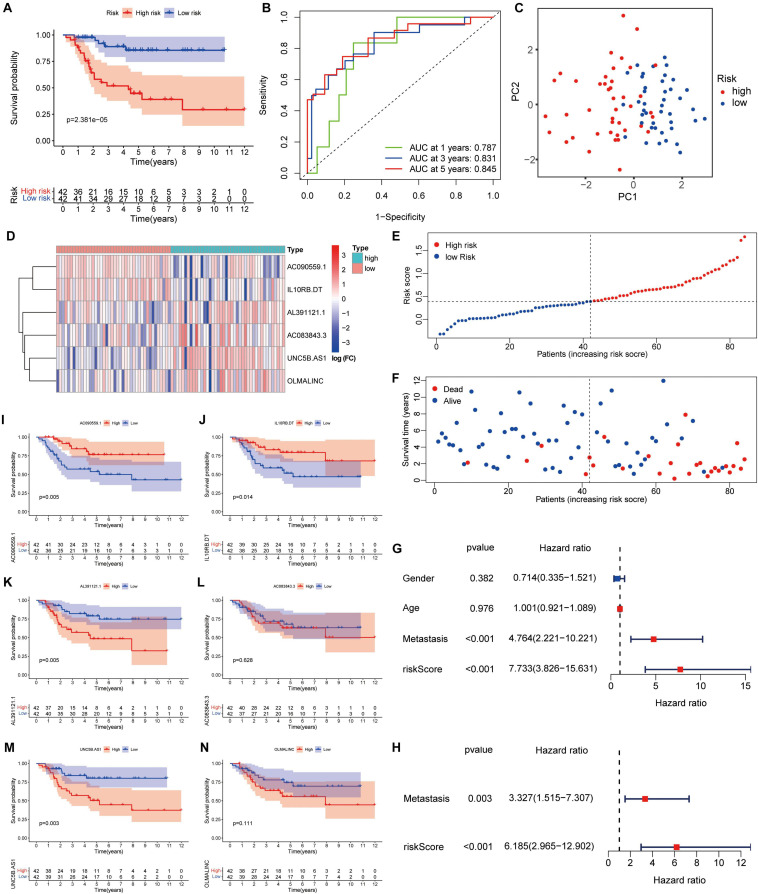
A risk signature with the six autophagy-related lncRNA. **(A)** Kaplan–Meier survival curve analysis. **(B)** The AUC for the prediction of 1, 3, 5-year survival rate. **(C)** PCA based on the autophagy-related long noncoding RNAs (lncRNAs) signature. **(D)** Heatmap of the six key lncRNAs expressed between the high and low risk group. **(E,F)** Risk score analysis of the signature. **(G,H)** Univariate and multivariate cox analysis. **(I–N)** Kaplan–Meier survival curves for the six prognostic lncRNAs in TARGET dataset.

Survival analysis showed that high-risk patients had the worse outcomes (Figure 2A). According to the ROC analysis, the predictive performance of the ARLs signature for 1-, 3-, and 5-year survival of osteosarcoma patients was excellent, with AUC values of 0.813, 0.814, and 0.802, respectively (Figure 2B). The PCA revealed different distribution patterns for the two groups of patients (Figure 2C). The risk score distribution, survival status, and six ARLs heat map were obtained (Figures 2D–F). The univariate Cox and multivariate Cox analyses showed that metastasis and risk score were significantly correlated with overall survival (Figures 2G,H). The risk score of patients with metastasis tended to be higher (Supplementary Figure 1C), although the difference was not significant (*p* = 0.071). In the Kaplan–Meier curves for single genes, the expression of AL391121.1 and UNC5B.AS1 was negatively correlated with prognosis of osteosarcoma patients, whereas the expression of AC090559.1, and IL10RB.DT was positively correlated (Figures 2I–N).

### Gene Set Enrichment Analysis and Immune Score Analysis

**FIGURE 3:**
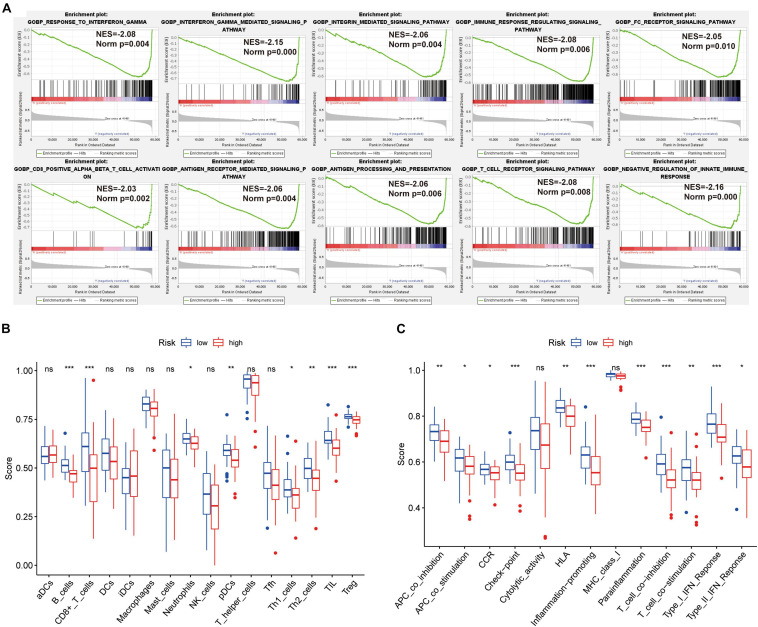
GSEA of osteosarcoma patients based on the autophagy-related lncRNA prognostic signature. **(A)** GSEA results show significant enrichment of immune- and autophagy-related biological process in the low-risk patients. **(B,C)** Relationship between riskscore and immune cell infiltration and related functions *via* ssGSEA analysis. The score refers to the immune score, the higher the score, and the deeper the degree of immune cell infiltration. *P < 0.05, **P < 0.01, and ***P < 0.001.

The GSEA revealed that immune-related biological processes were significantly enriched in the low-risk group, including the T cell receptor-signaling pathway, integrin mediated-signaling pathway, and response to interferon gamma (Figure 3A). This result suggested that activation of immunomodulatory functions in the low-risk group led to a better prognosis. On the basis of ssGSEA, the correlations between the risk score and immune cells and immune-related functions were determined, and as shown in the boxplots in Figures 3B,C, there were significant differences between low-risk and high-risk groups for B cells, CD8^+^ T cells, TIL, Treg, immune checkpoint, regulation of inflammation, and T cell co-inhibition.

### Differentially Expression of ARGs and Functional Analysis

**FIGURE 4:**
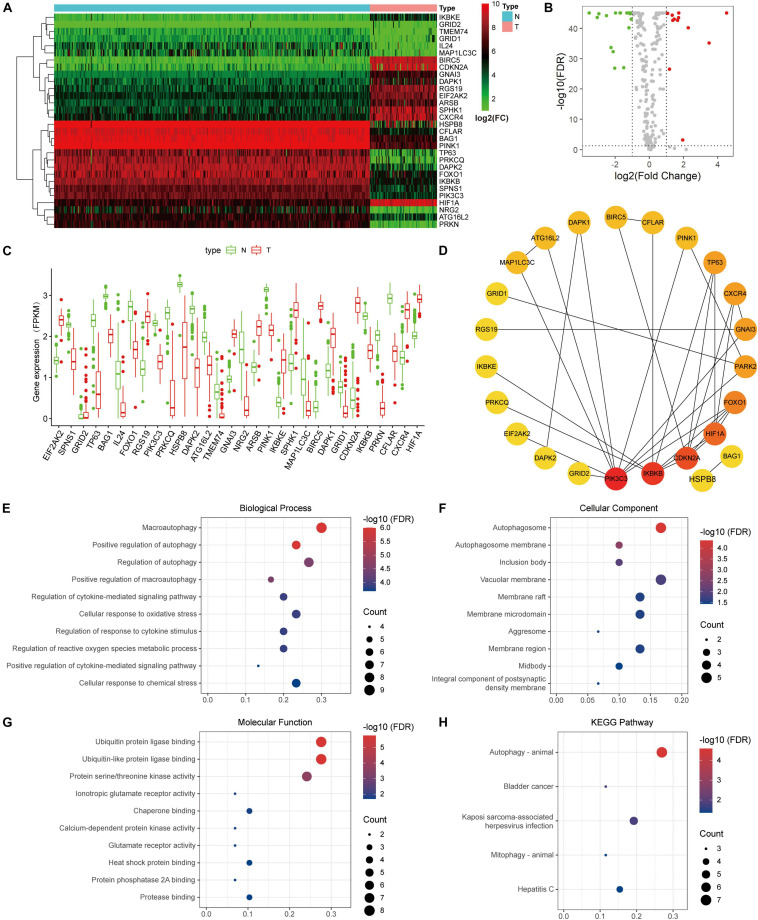
Differentially expressed autophagy-related genes (ARGs) and functional enrichment. **(A)** Heatmap of the differentially expression ARGs. **(B)** Volcano plot. **(C)** The expression patterns of 30 ARGs in osteosarcoma and normal samples. The *X*-axis represents the differentially expressed ARGs and the *Y*-axis represents their gene expression levels (FPKM). **(D)** Protein-protein interaction network of the ARGs. **(E–G)** Functional enrichment of the 30 ARGs including biological process, cellular component and molecular function. **(H)** KEGG pathway analysis.

Thirty DE-ARGs were detected, of which 12 were up-regulated and 18 were down-regulated. The DE-ARGs between osteosarcoma and healthy tissues are shown in a heat map, and volcano plot in Figures 4A,B. The expression levels of the 30 DE-ARGs between the two groups were displayed with box plots (Figure 4C). The PPI network illustrated the relations between the DE-ARGs (Figure 4D).

To elucidate the underlying mechanisms of the DE-ARGs, functional and pathway enrichment analyses were used. In biological processes, the DE-ARGs primarily participated in macroautophagy and regulation of autophagy, and macroautophagy (Figure 4E). In molecular functions, enriched terms were primarily related to ubiquitin protein ligase binding, ionotropic glutamate receptor activity, and protein serine/threonine kinase activity (Figure 4F). In cellular components, enriched terms were primarily related to autophagosome, inclusion body, and vacuolar membrane (Figure 4G). KEGG analysis revealed that the DE-ARGs were mainly enriched in autophagy-animal pathways (Figure 4H).

### Establishment of ARGs Prognostic Signature

**FIGURE 5:**
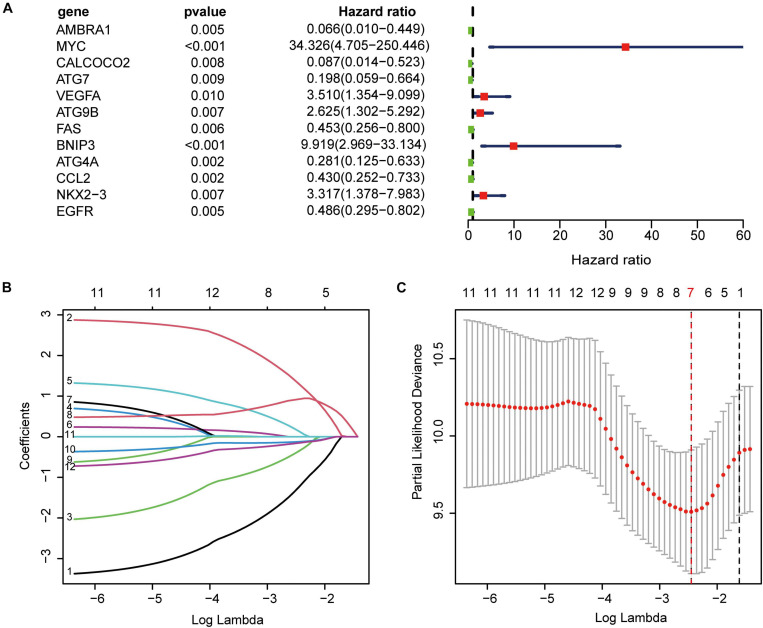
Construction of the autophagy-related prognostic signature. **(A)** Univariate cox regression showed 12 ARGs related to osteosarcoma survival. **(B)** LASSO coefficient spectrum of the 12 genes. Coefficients refers to the risk coefficient corresponding to each gene. **(C)** Selection of the optimal λ value.

Using univariate Cox regression analysis for the 222 ARGs, we identified 12 prognostic ARGs (Figure 5A). To construct the signature, LASSO Cox regression analysis was performed to these 12 ARGs, from which seven genes were selected (Figures 5B,C). At last, we used multivariate Cox regression analysis to build a ARGs prognostic signature, and three genes and their risk coefficients were identified: AMBRA1, MYC, and VEGFA (Supplementary Table 3).

### Evaluation of the ARGs Prognostic Signature

**FIGURE 6:**
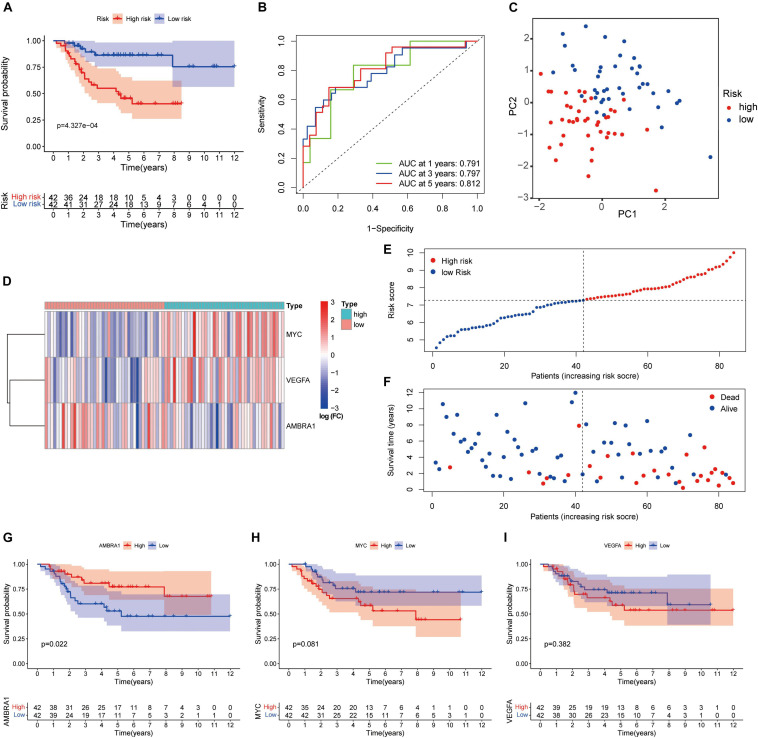
Autophagy-related genes (ARGs) signature based on TARGET. **(A)** Kaplan–Meier curves result. **(B)** The AUC for the prediction of 1, 3, 5-year survival rate of osteosarcoma. **(C)** PCA based on the confirmed three ARGs signature. **(D)** Heatmap of the key genes expressed between the high and low risk group. **(E,F)** Risk score analysis of the signature. **(G–I)** Kaplan–Meier curves results of single gene.

We performed survival analysis to evaluate the signature. The Kaplan–Meier curve revealed that high-risk patients were accompanied by poor prognosis (Figure 6A). The ROC curve showed the prediction capability of the ARGs prognostic signature for 1-, 3- and 5-year survival rates in the TARGET cohort. The curve (AUC) was 0.791, 0.797, and 0.812, respectively (Figure 6B). Principal component analysis (PCA) showed the different distribution patterns for the two groups of patients (Figure 6C). The heatmap shows the expression profiles of these key genes (Figure 6D). The dot plot shows the distribution of individual patients (Figures 6E,F). In order to understand the role of these three prognostic genes in osteosarcoma, and Kaplan–Meier curve was drawn for them. AMBRA1 is significantly associated to the survival of osteosarcoma and plays a protective role (Figures 6G–I).

### Clinical Relevance of the ARGs Prognostic Signature

**FIGURE 7:**
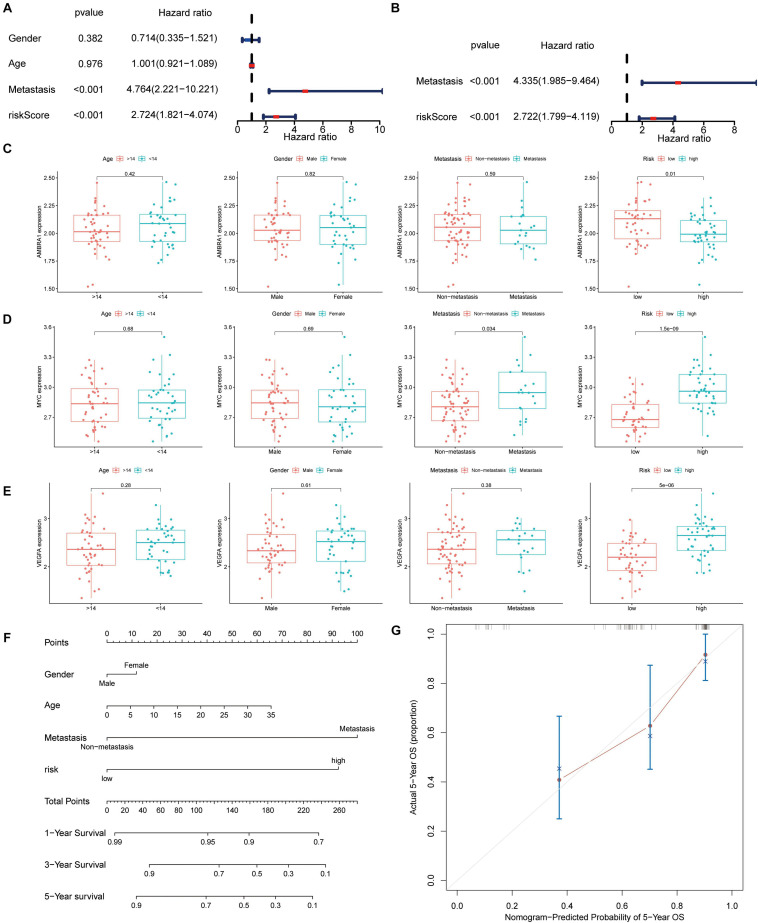
Autophagy-associated gene signature was significantly related to survival in osteosarcoma and clinical correlation analyses. **(A,B)** Univariate and multivariate cox analysis showed the autophagy-related genes signature and metastasis were two independent predictor of prognosis in osteosarcoma. **(C–E)** The expression of the three key genes, the patients from the TARGET database were grouped according to age (< 14 y; ≥ 14 y), gender, metastasis, and riskscore. The *X*-axis represents the different groups and the *Y*-axis represents their gene expression levels (FPKM). **(F)** Nomogram based on gender, age, metastasis, and risk in the TARGET database. **(G)** Calibration plots of the nomogram for predicting the probability of OS at 5 years.

The univariate and multivariate Cox regression analysis results revealed that the signature and tumor metastasis were two independent prognostic factors (Figures 7A,B). The relationship between the three key genes and clinical characteristics was displayed with box plot, it shows that in the low-risk patients, and the expression level of AMBRA1 is highly (Figure 7C). However, the opposite trend was found for the expression of MYC and VEGFR (Figures 7D,E). It is worth noting that only MYC was associated with metastasis, and that it was highly expressed in patients of the metastatic group (Figure 7D). And we attempted to determine whether the metastatic status was associated with the risk score, calculated based on the autophagy related genes prognostic signature (ARGs signature). The results revealed that osteosarcoma patients with metastasis (Supplementary Figure 1A) had higher risk scores, and it has been verified in the GSE21257 data set (Supplementary Figure 1B). By multivariate Cox regression, we built a nomogram model that contains indicators such as gender, age, metastasis, and risk (Figure 7F). The calibration curve shows that the nomogram has high prediction accuracy in the 3 and 5 years outcomes (Figure 7G and Supplementary Figure 1C), and it also shows good osteosarcoma prediction accuracy in the validation cohort (Supplementary Figures 1D,E).

### Verification of the ARGs Signature in Dataset GSE21257

**FIGURE 8:**
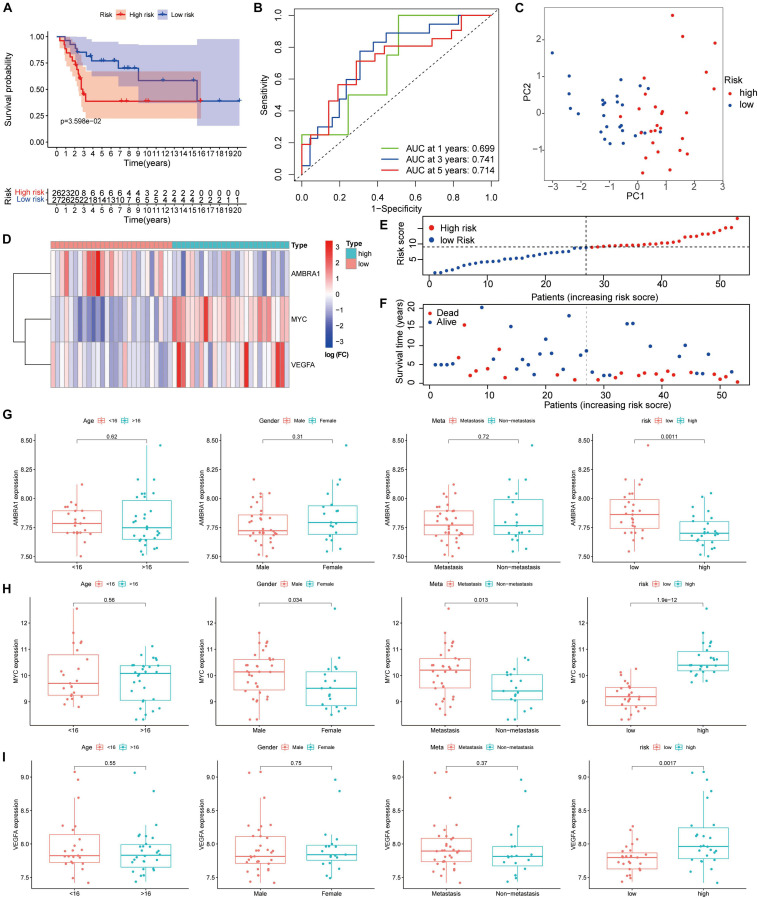
Validation of the autophagy-associated gene signature in the testing group. **(A)** Kaplan–Meier curves result. **(B)** The AUC for the prediction of 1, 3, 5-year survival rate. **(C)** PCA based on the three autophagy-related genes signature. **(D)** Heatmap of the three genes expressed between the high and low risk group. **(E,F)** Risk score analysis of the signature. **(G–I)** The expression of the three key genes, the patients from the GSE21257 dataset were grouped according to age (< 16 y; ≥ 16 y), gender, metastasis, and riskscore. The *X*-axis represents the different groups and the *Y*-axis represents their gene expression levels (normalized signal values).

We verified the results in the GSE21257 dataset using the same risk coefficient. Consistent with the results from the TARGET database, in comparison between the two groups, and the high-risk patients tend to have poorer outcomes (Figure 8A). As shown by the ROC curve, the 1, 3, and 5-year AUC of the survival rate was 0.699, 0.741, and 0.714, respectively in the GSE21257 cohort (Figure 8B). Similarly, based on the risk coefficient of three key genes, we conducted PCA (Figure 8C). The heatmap shows the expression profile of ARGs in the GSE21257 dataset (Figure 8D), and the dot plot shows the distribution of prognostic indicators and the survival status of different groups of patients (Figures 8E,F). Based on these results, we confirmed that the prognosis of osteosarcoma patients can be reliable predicted by the ARGs signature. Moreover, the box plot shows that the relationship between the key genes and clinical information is consistent with the results obtained from the TARGET dataset (Figures 8G–I).

### Correlation Between the Expression Levels of Autophagy-Related Prognostic Markers

**FIGURE 9:**
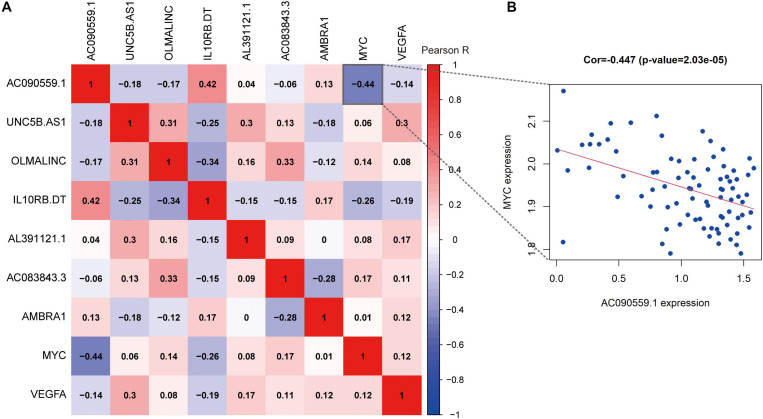
The correlation analysis of prognostic autophagy-associated biomarkers. **(A)** The *x*-axis and *y*-axis represent genes. Red blocks represent positive correlation, and blue blocks represent negative correlation. And shade of color represents Pearson correlation index R. **(B)** Correlation between the expression of lncRNA AC090559.1 and MYC gene expression.

To better understand the correlations among the ARLs and ARGs, we applied the Pearson correlation analysis. We found that the expression of lncRNA AC090559.1 is positively correlated with the expression of IL10RB.DT and negatively associated with MYC expression. The results shown in Figure 9.

### Verification the Expression Level of Autophagy-Related Markers

**FIGURE 10:**
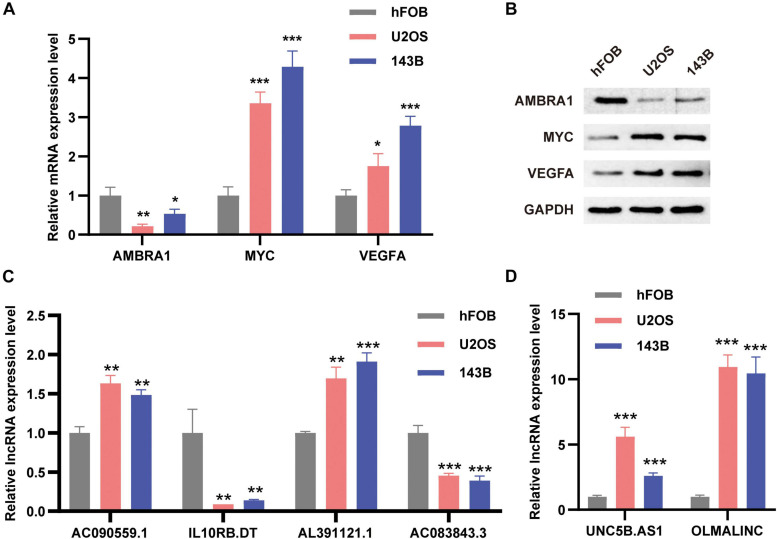
The expression levels of ARGs and ARLs in osteosarcoma cell lines were compared with those of osteoblasts. **(A)** The qRT-PCR result of the three ARGs was evaluated by the 2–ΔΔCT method. **(B)** Western blotting results of AMBRA1, MYC, and VEGFA expression. **(C,D)** The qRT-PCR result of the six ARLs was evaluated by the 2-ΔΔCT method. The data are expressed as mean ± standard deviation. *P < 0.05, **P < 0.01, and ***P < 0.001.

In order to verify the expression levels of autophagy-related prognostic markers, we examined the expression levels of the three ARGs and the six ARLs in the osteoblast cell line hFOB and two osteosarcoma cell lines (U2OS and 143B) by using qRT-PCR and Western blotting analysis. Our results showed that compared with osteoblasts, AMBRA1 was down-regulated in U2OS and 143B, and while MYC and VEGFA were up-regulated in osteosarcoma cells (Figures 10A,B). For lncRNAs, AC090559.1, AL391121.1, UNC5B.AS1, and OLMALINC were significantly up-regulated in 143B and U2OS cell lines, while IL10RB.DT and AC083843.3 was down-regulated (Figures 10C,D).

## Discussion

Osteosarcoma is a common primary malignant bone tumor, and it remains a disease with high mortality rates in children and adolescents (Jo and Fletcher, 2014). Over the past 40 years, progress in the treatment of osteosarcoma has plateaued (Ballatori and Hinds, 2016), and innovative and effective strategies to overcome the current limitations are urgently needed. Autophagy is associated with the progression of osteosarcoma and may be related to the emergence of drug resistance during treatment (Niu et al., 2019). However, most studies examine the correlation between a single ARG and osteosarcoma (Liu et al., 2017; Livingston et al., 2018; Zhao et al., 2018), and the characteristics of ARGs in patients with osteosarcoma and their correlation with overall survival have not been systematically evaluated.

Because some lncRNAs can regulate tumor progression and metastasis by targeting ARGs, they can be used as prognostic biomarkers of cancer (Chen et al., 2017; Huang et al., 2018). In the analysis of public RNA sequence data in this study, 13 prognostic ARLs were identified. LASSO Cox regression analysis indicated that six ARLs (AC090559.1, IL10RB.DT, AL391121.1, AC083843.3, UNC5B.AS1, and OLMALINC) were good candidates to be prognostic markers. As shown in the autophagy-related lncRNA model, high-risk patients had significantly poorer outcomes than those of low-risk patients. The prognostic signature based on the six ARLs was also independent of other clinical characteristics. GSEA functional enrichment analysis showed that the low-risk group was enriched in some pathways related to autophagy and immune regulation, which indicated that immunomodulation was related to an improved prognosis. We also discussed the immune-related characteristics. In general, the degree of immune cell infiltration decreased significantly in the high-risk patients, and immune-related functions, including regulation of checkpoint, inflammation, and T cell co-inhibition, were also down-regulated significantly. These results indicate that high-risk patients have systemic immunosuppression, which may affect their prognosis. Thus, the six prognostic ARLs could accurately predict the overall survival in osteosarcoma. Notably, Wu et al. (2021) recently constructed an autophagy-related lncRNA signature, which also includes AC090559.1, that can predict the prognosis of lung adenocarcinoma. The ARLs IL10RB-DT and AL391121.1 are associated with the prognosis of clear cell renal cell carcinoma (Jiang et al., 2020; Yang et al., 2021), and compared with normal human renal cell lines, IL10RB-DT is highly expressed in renal cancer cell lines (Jiang et al., 2020). The ARL UNC5B.AS1 is highly expressed in a variety of tumors (Wang Y. et al., 2019; Tan J.J. et al., 2020; Tan S.F. et al., 2020; Wang et al., 2020), and is important in tumor proliferation and progression. In addition, In addition, Tan S.F. et al. (2020), showed that UNC5B-AS1 promotes the malignant progression of prostate cancer by competing with caspase-9, and caspase-9 is closely related to autophagy (An et al., 2020). In this study, UNC5B.AS1 was significantly up-regulated in osteosarcoma cell lines, compared with osteoblasts. Thus, UNC5B-AS1 also appears to be important in osteosarcoma, possibly *via* its association with autophagy. In this study, OLMALINC expression was also significantly higher in osteosarcoma cell lines. The role of OLMALINC in tumors has not been reported, but the results in this study indicate that further study of its functions is warranted.

Autophagy is an intracellular metabolic process that relies on the lysosomal degradation of cytoplasmic proteins, organelles, and pathogens (Kim and Lee, 2014). It is important to the dynamic balance of cells, tissues, and organisms, and is mediated by evolutionarily conserved ARGs (Levine and Kroemer, 2019). In cancer, autophagy may be inhibitory or stimulatory, depending on the type, stage, or genetic background of the disease. In this study, 30 DE-ARGs were identified between osteosarcoma and healthy tissues. Functional enrichment analysis of the DE-ARGs revealed that GO terms or signal pathways related to autophagy were enriched in osteosarcoma tissue. Univariate Cox regression analysis was used to identify 12 prognostic ARGs, and then, LASSO Cox regression analysis was used to identify the seven genes that were the best candidates. Last, multivariate regression analysis was used to complete the prognostic signature composed of three ARGs (AMBRA1, MYC, and VEGFA). Based on the risk coefficients, risk scores were obtained, and the patients were grouped. Survival analysis showed that high-risk patients evolved with poor prognosis. In addition, univariate and multivariate Cox regression analyses indicated that the signature and tumor metastasis were two independent prognostic factors. More importantly, the independent data set GSE21257 was used to validate the ARGs prognostic signature.

Currently, the most widely studied nuclear oncogene is MYC (Dang, 2012). Chen D. et al. (2018) revealed that MYC expression level increases significantly in samples of a metastatic group, compared with that in a non-metastatic group, and indicating that increased expression of MYC may be related to osteosarcoma metastasis. In addition, the expression of MYC in osteosarcoma samples is inversely proportional to survival time (Chen D. et al., 2018). These results are consistent with those in this study. Vascular endothelial growth factor-A (VEGFA, also known as VEGF) is a highly specific pro-vascular endothelial cell growth factor that is overexpressed in most human tumors, and its expression is related to tumor aggressiveness, increased blood vessel density, metastasis, tumor recurrence, and poor prognosis (Claesson-Welsh and Welsh, 2013; Ferrara and Adamis, 2016). Wu et al. (2019) found that VEGF is highly expressed in osteosarcoma tissues and that high VEGF expression is significantly correlated with low overall survival rate. AMBRA1 is an autophagy regulatory protein, and Cecconi et al. (Fimia et al., 2007; Nazio et al., 2013; Cianfanelli et al., 2015) showed that it may be associated with cell proliferation, tumorigenesis, and neurodevelopment. In this study, AMBRA1 was closely related to the prognosis of osteosarcoma. However, its specific mechanism of action in osteosarcoma has not been reported. Therefore, how AMBRA1 may be involved in the pathology of osteosarcoma requires additional investigation.

A variety of autophagy-related prognostic models have been established. For example, Qiu et al. (2020) analyzed the RNA-seq data of 375 hepatocellular carcinoma patients from TCGA and obtained a four-gene signature that has predictive prognosis value. Wang S.S. et al. (2019) also proposed a four-gene prognostic marker as a potential survival prediction marker for bladder cancer patients. However, the results of those studies have not been verified in experiments. In this study, the TARGET tumor data set and the normal tissue GTEx data set were combined to establish a prognostic model of autophagy-related markers in osteosarcoma for the first time. The model was verified using an independent database, and its reliability was demonstrated in an experiment. Although a prognostic model was successfully developed with the approach used in this study, there were also some limitations. First, the number of healthy (*n* = 396) and osteosarcoma (*n* = 84) tissue samples was very different in the respective publicly available GTEx and TARGET databases, and which might distort the results. Thus, additional tumor samples need to be analyzed in the future. Second, because of the lack of lncRNA expression data, autophagy-related lncRNA models could not be verified in external data sets.

In summary, our research has shown that prognostic signatures comprising ARGs and ARLs can exactly predict the osteosarcoma overall survival. Moreover, our work highlights the importance of autophagy-related markers in osteosarcoma, and proposes the exploration of ARGs or ARLs as prospective biomarkers in osteosarcoma therapy.

## Data Availability Statement

The datasets presented in this study can be found in online repositories. The names of the repository/repositories and accession number(s) can be found below: The datasets analyzed for this study can be found in the TARGET (https://portal.gdc.cancer.gov/), Project ID: TARGET-OS, Gene Expression Omnibus (GEO) database (https://www.ncbi.nlm.nih.gov/geo/), accession number:GSE21257, Genotype-Tissue Expression (GTEx) database (https://gtexportal.org/).

## Author Contributions

JZ and XC designed the study. RD and TW searched the data from database. JZ and TW performed analysis of the data. JZ and RD carried out the experiments and analyzed the experimental results, and wrote the original draft of the manuscript. JJ and XC supervised this work revised the manuscript. All authors had read and approved the final manuscript.

## Conflict of Interest

The authors declare that the research was conducted in the absence of any commercial or financial relationships that could be construed as a potential conflict of interest.

## Publisher’s Note

All claims expressed in this article are solely those of the authors and do not necessarily represent those of their affiliated organizations, or those of the publisher, the editors and the reviewers. Any product that may be evaluated in this article, or claim that may be made by its manufacturer, is not guaranteed or endorsed by the publisher.

## Footnotes

1
https://gtexportal.org/

2
https://portal.gdc.cancer.gov/

3
https://www.ncbi.nlm.nih.gov/geo/

4
http://www.autophagy.lu/

## Supplementary Material

Supplementary Figure 1 **(A–C)** The relationship between autophagy-related markers and metastasis. **(D–F)** The calibration curve of the nomogram.

Supplementary Table 1 Primers used in this study.

Supplementary Table 2 Risk coefficients of six ARLs.

Supplementary Table 3 Risk coefficients of three ARGs.
