# Supplementary figures and images for: Autophagy-Related Genes and Long Noncoding RNAs Signatures as Predictive Biomarkers for Osteosarcoma Survival
Source: Front Cell Dev Biol. 2021 Aug 26;9:705291. doi: 10.3389/fcell.2021.705291 (PMC8427445; doi:10.3389/fcell.2021.705291)

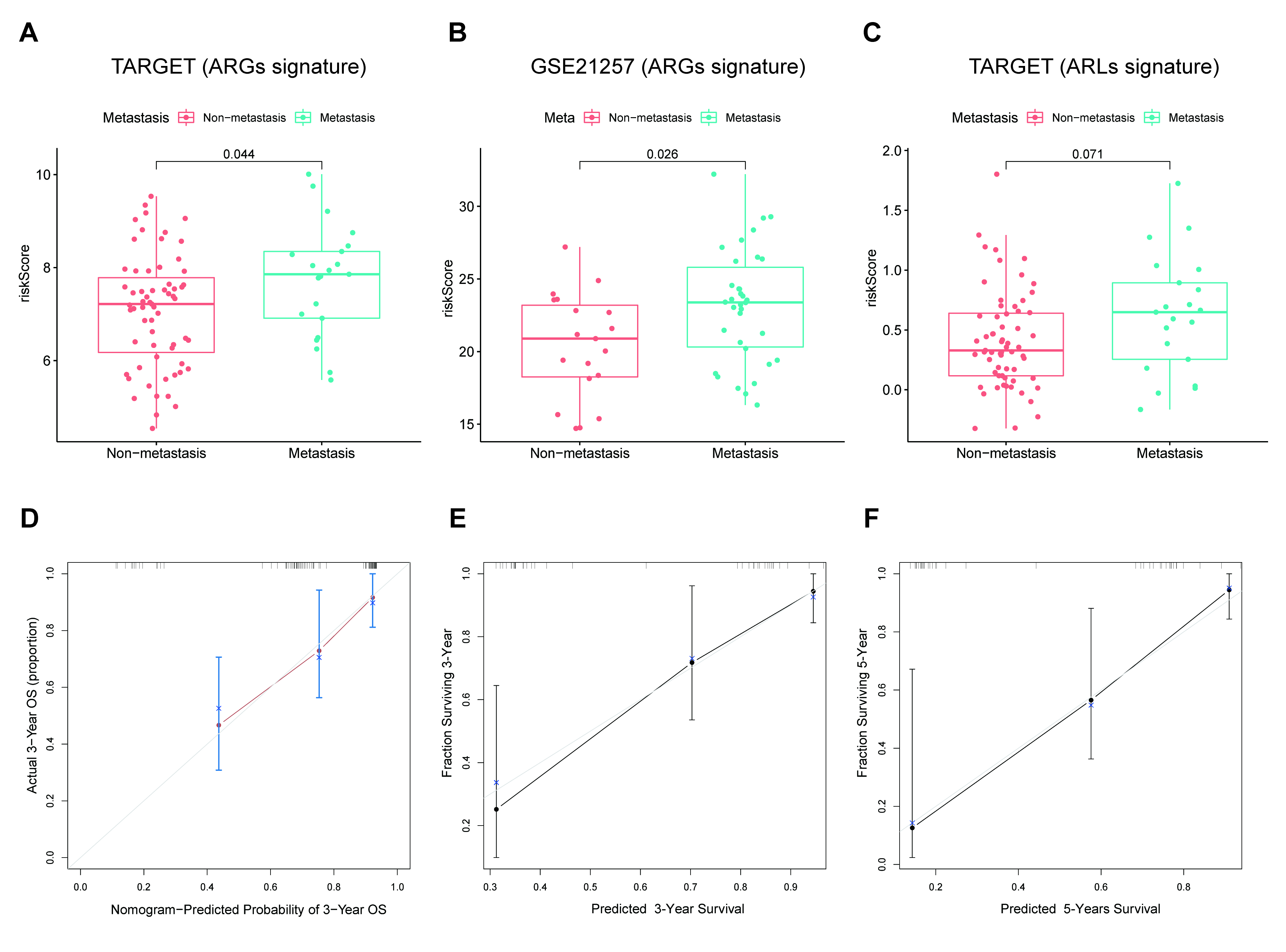

Supplement: Supplementary Figure 1 — (A–C) The relationship between autophagy-related markers and metastasis. (D–F) The calibration curve of the nomogram. [file Image_1.TIF]
