# Supplementary material for: Autophagy-Related Genes and Long Noncoding RNAs Signatures as Predictive Biomarkers for Osteosarcoma Survival
Source: Front Cell Dev Biol. 2021 Aug 26;9:705291. doi: 10.3389/fcell.2021.705291 (PMC8427445; doi:10.3389/fcell.2021.705291)
Supplement: Supplementary Table 1 — Primers used in this study. [file Table_1.docx]

**Supplementary Table 1. Primers used in this study.**

| Gene | Direction | Sequences (5’ to 3’) |
| --- | --- | --- |
| AMBRA1 | Forward | AACCCTCCACTGCGAGTTGA |
|  | Reverse | TCTACCTGTTCCGTGGTTCTCC |
| MYC | Forward | GGGTAGTGGAAAACCAGCAGCCTC |
|  | Reverse | CATCTTCTTGTTCCTCCTCAGAGTCGC |
| VEGFA | Forward | GCCTTGCCTTGCTGCTCTAC |
|  | Reverse | TGATTCTGCCCTCCTCCTTCTG |
| GAPDH | Forward | GAAGGTCGGAGTCAACGG ATTTG |
|  | Reverse | ATGGCATGGACTGTGGTCATGAG |
| AC090559.1 | Forward | ACTAATCAGGGGCACTGATGAAG |
|  | Reverse | AATCTTGGACGCTCTAGGGACTT |
| IL10RB.DT | Forward | CTTTCAGTATTCCCCAGGTTTTG |
|  | Reverse | ACTTCCCTCTGCTTGACTTTACG |
| AL391121.1 | Forward | GTCATTCAGATGAAATTCCAGTCC |
|  | Reverse | TCCGTTTCTATTTGTGAAGGGAG |
| AC083843.3 | Forward | GATTGATTACATCATTGGTCGCC |
|  | Reverse | ATTTGCTCTTCATCTCCATTTCC |
| UNC5B.AS1 | Forward | GATCCTGCCTCAGGGAAA |
|  | Reverse | GCTCAAGAGGTTGGGACT |
| OLMALINC | Forward | GACTCCTTTGGGAGACCAGTG |
|  | Reverse | AGGTCACAGGGGATTTGATGG |

**Supplementary Table 2. Risk coefficients of six ARLs.**

| Gene | Coefient |
| --- | --- |
| AC090559.1 | -0.328103908459367 |
| UNC5B.AS1 | 0.40585590034141 |
| OLMALINC | 0.00137290934158311 |
| IL10RB.DT | -0.338699939795244 |
| AL391121.1 | 0.258628116479713 |
| AC083843.3 | 0.102484560997761 |

ARLs: autophagy-related lncRNAs.

| Symbol | Coeffient | HR | HR.95L | HR.95H | pvalue |
| --- | --- | --- | --- | --- | --- |
| AMBRA1 | -3.4704 | 0.0311 | 0.0024 | 0.3983 | 0.0076 |
| MYC | 3.8230 | 45.7406 | 4.5574 | 459.0747 | 0.0012 |
| VEGFA | 1.4389 | 4.2157 | 1.5724 | 11.3024 | 0.0042 |

**Supplementary Table 3. Risk coefficients of three ARGs.**

ARGs: autophagy-related genes.
